# μ-Oxido-bis­[bis­(phenanthroline-κ^2^ *N*,*N*′)(sulfato-κ*O*)iron(III)] octa­hydrate

**DOI:** 10.1107/S1600536811042723

**Published:** 2011-10-22

**Authors:** JingYa Zhang, Ling Wang, Yanju Liu

**Affiliations:** a Pharmacy College, Henan University of Traditional Chinese Medicine, Zhengzhou 450008, People’s Republic of China; b Chemistry Department, Zheng Zhou Normal University, Zhengzhou 450044, People’s Republic of China

## Abstract

The title complex, [Fe_2_O(SO_4_)_2_(C_12_H_8_N_2_)_4_]·8H_2_O, contains two unique Fe^III^ cations, one oxide anion, four 1,10-phenanthroline (phen) ligands, two coordinated sulfate anions and eight lattice water mol­ecules. Each Fe^III^ ion has an approximate octa­hedral geometry, coordinated by four N atoms from two phen mol­ecules, two O atoms from oxide and sulfate anions, respectively. The parallel phen mol­ecules form two-dimensional supermolecules through π–π stacking inter­actions [centroid–centroid distances = 3.684 (3), 3.711 (3), 3.790 (3), 3.847 (3), 3.746 (3), 3.732 (3) and 3.729 (3) Å]. This architecture is further stabilized by O—H⋯O hydrogen bonds involving the lattice water mol­ecules and sulfate O atoms.

## Related literature

For transition metal complexes containing organic ligands with nitro­gen heteroatoms, see: Manson *et al.* (2001 ▶); Wu *et al.* (2009 ▶); Accorsi *et al.* (2009 ▶); Xie & Huang (2011 ▶); Feng *et al.* (2006 ▶); Yu *et al.* (2010 ▶); Weyhermüller *et al.* (2005 ▶). For phen (1,10-phenanthroline) ligands, see: Gu *et al.* (2006 ▶); Hu *et al.* (2009 ▶). For related bond lengths and angles, see: Yang *et al.* (2010 ▶).
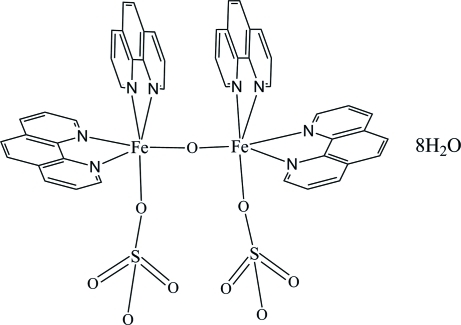


## Experimental

### 

#### Crystal data

[Fe_2_O(SO_4_)_2_(C_12_H_8_N_2_)_4_]·8H_2_O
*M* *_r_* = 1184.76
Monoclinic, 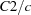

*a* = 21.589 (15) Å
*b* = 14.181 (10) Å
*c* = 16.500 (12) Å
β = 97.289 (9)°
*V* = 5010 (6) Å^3^
*Z* = 4
Mo *K*α radiation
μ = 0.75 mm^−1^
*T* = 273 K
0.20 × 0.10 × 0.04 mm

#### Data collection

Bruker APEXII CCD area-detector diffractometer
Absorption correction: multi-scan (*SADABS*; Sheldrick, 1995 ▶) *T*_min_ = 0.865, *T*_max_ = 0.971
11655 measured reflections
4398 independent reflections
3506 reflections with *I* > 2σ(*I*)
*R*_int_ = 0.029

#### Refinement

*R*[*F* ^2^ > 2σ(*F* ^2^)] = 0.037
*wR*(*F* ^2^) = 0.107
*S* = 1.05
4398 reflections
372 parameters
15 restraints
H atoms treated by a mixture of independent and constrained refinement
Δρ_max_ = 0.66 e Å^−3^
Δρ_min_ = −0.34 e Å^−3^

### 

Data collection: *APEX2* (Bruker, 2007 ▶); cell refinement: *SAINT* (Bruker, 2007 ▶); data reduction: *SAINT*; program(s) used to solve structure: *SHELXS97* (Sheldrick, 2008 ▶); program(s) used to refine structure: *SHELXL97* (Sheldrick, 2008 ▶); molecular graphics: *SHELXTL* (Sheldrick, 2008 ▶); software used to prepare material for publication: *SHELXTL*.

## Supplementary Material

Crystal structure: contains datablock(s) global, I. DOI: 10.1107/S1600536811042723/jj2103sup1.cif

Structure factors: contains datablock(s) I. DOI: 10.1107/S1600536811042723/jj2103Isup2.hkl

Additional supplementary materials:  crystallographic information; 3D view; checkCIF report

## Figures and Tables

**Table 1:** Selected geometric parameters (Å, °)

Fe1—O1	1.7804 (10)
Fe1—O2	1.936 (2)
Fe1—N4	2.125 (2)
Fe1—N1	2.151 (2)
Fe1—N3	2.237 (3)
Fe1—N2	2.243 (2)

**Table 2:** Hydrogen-bond geometry (Å, °)

*D*—H⋯*A*	*D*—H	H⋯*A*	*D*⋯*A*	*D*—H⋯*A*
O3*W*—H3*WA*⋯O3^i^	0.85 (1)	2.14 (3)	2.872 (4)	144 (4)
O2*W*—H2*WB*⋯O4^ii^	0.85 (1)	1.89 (2)	2.713 (4)	163 (4)
O4*W*—H4*WB*⋯O5	0.85 (1)	1.98 (2)	2.756 (4)	151 (4)
O1*W*—H1*WB*⋯O3*W*^iii^	0.86 (1)	2.06 (1)	2.909 (5)	171 (4)
O1*W*—H1*WA*⋯O4*W*^iv^	0.86 (1)	1.97 (2)	2.811 (5)	165 (5)
O3*W*—H3*WB*⋯O4*W*^v^	0.85 (1)	2.28 (3)	2.964 (5)	138 (3)

Symmetry codes: (i) 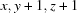; (ii) 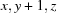; (iii) 
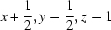
; (iv) 
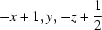
; (v) 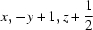.

**Fig. 1.**
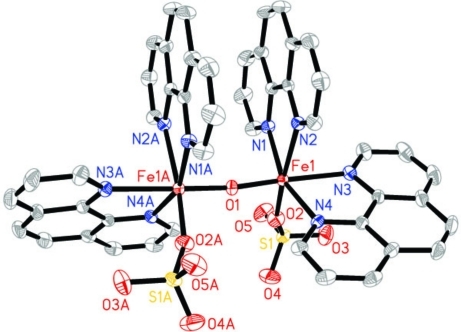
The molecular structure for title compound. Displacement ellipsoids at the 30% probability level. Hydrogen atoms have been deleated for clarity.

## supplementary crystallographic information

### Comment

Organic ligands containing nitrogen heteroatoms play an important role in the assembling process of transition-metal complexes (Manson *et al.*, 2001; Wu *et al.*, 2009; Accorsi *et al.*, 2009; Xie *et al.*, 2011; Feng *et al.*, 2006; Yu *et al.*, 2010; Weyhermuller *et al.*, 2005). Phen (1,10-phenanthroline) ligands fit together to form transition-metal complexes (Gu *et al.*, 2006; Hu *et al.*, 2009). In order to study the coordination behavior of this ligand to Fe, we have synthesized herein the title complex [(Fe_2_O)(phen)_4_(SO_4_)_2_).8H_2_O], (I).  The asymmetric unit contains one Fe^III^ atom, one half of an O^2-^ atom, two phen ligands, one coordinated SO_4_ anion and four lattice water molecules (Fig. 1). The phen ligands lie parallel to each other in the structure and form two-dimensional supermolecules through π–π stacking inteactions [centroid–centroid distances = 3.684 (3)Å (Cg1—Cg1)^i^; 3.711 (3)Å (Cg3—Cg4)^i^, 3.790 (3)Å; (Cg4—Cg4)^i^, 3.847 (3)Å (Cg4—Cg7)^i^; 3.746 (3)Å (Cg6—Cg6)^ii^; 3.732(3(Å (Cg7—Cg7)^i^ and 3.729(30Å Cg8—Cg6)^ii^ where *i* = 1-x, y, 1/2-z; *ii* = 1-x, -y, -z and Cg1 = Fe/N1/C5/C10/N2; Cg3 = Fe1/N3/C17/C22/N4; Cg4 = N2/C6–C10;  Cg6 = N4/C18–C22; Cg7 = C4/C5/C9–C12;  Cg8 = C16/C17/C21–C24].  This architecture is further stabilized by O—H···O hydrogen bonds involving the lattice water molecules and oxygen atoms from the SO_4_ anions (Table 1). The bond distances for Fe—N vary from 2.125 (2)Å to 2.243 (2)Å, and the angles for N—Fe—N and N—Fe—O are between 75.21 (10)° and 168.95 (6)°, respectively. The Fe—O bond lengths are 1.7804 (10)Å, 1.936 (2)Å and the bond angle for O1—Fe—O2 is 97.99 (10)°, respectively. These bond distances and bondangles are in agreement with those found in the reported iron phen compounds (Yang *et al.* 2010).

### Experimental

0.151 g of 1,10-phenanthroline hydrate was dissolved in methanol (5 ml). To the solution, 5 ml of H_2_O was added, then layered with 5 ml of a methanol solution of Fe_2_(SO_4_)_3_ (0.020 g). The resulting solution was allowed to stand at room temperature for several days and black block crystals were obtained.

### Refinement

Water H atoms were located in a difference Fourier map and refined isotropically with restrained O—H distance = 0.85 Å and an H···H distance = 1.37 Å. The remaining H atoms were generated geometrically and then refined using the riding model with C—H = 0.93 Å and *U*_iso_(H) = 1.2*U*_eq_(C).

### Crystal data

**Table:**

[Fe_2_O(SO_4_)_2_(C_12_H_8_N_2_)_4_]·8H_2_O	*F*(000) = 2448
*M**_r_* = 1184.76	*D*_x_ = 1.571 Mg m^−^^3^
Monoclinic, *C*2/*c*	Mo *K*α radiation, λ = 0.71073 Å
Hall symbol: -C 2yc	Cell parameters from 5306 reflections
*a* = 21.589 (15) Å	θ = 2.2–27.3°
*b* = 14.181 (10) Å	µ = 0.75 mm^−^^1^
*c* = 16.500 (12) Å	*T* = 273 K
β = 97.289 (9)°	Block, black
*V* = 5010 (6) Å^3^	0.20 × 0.10 × 0.04 mm
*Z* = 4	

### Data collection

**Table:**

Bruker APEXII CCD area-detector diffractometer	4398 independent reflections
Radiation source: fine-focus sealed tube	3506 reflections with *I* > 2σ(*I*)
graphite	*R*_int_ = 0.029
φ and ω scans	θ_max_ = 25.0°, θ_min_ = 2.1°
Absorption correction: multi-scan (*SADABS*; Sheldrick, 1995)	*h* = −25→24
*T*_min_ = 0.865, *T*_max_ = 0.971	*k* = −15→16
11655 measured reflections	*l* = −10→19

### Refinement

**Table:**

Refinement on *F*^2^	Primary atom site location: structure-invariant direct methods
Least-squares matrix: full	Secondary atom site location: difference Fourier map
*R*[*F*^2^ > 2σ(*F*^2^)] = 0.037	Hydrogen site location: inferred from neighbouring sites
*wR*(*F*^2^) = 0.107	H atoms treated by a mixture of independent and constrained refinement
*S* = 1.05	*w* = 1/[σ^2^(*F*_o_^2^) + (0.059*P*)^2^ + 4.8592*P*] where *P* = (*F*_o_^2^ + 2*F*_c_^2^)/3
4398 reflections	(Δ/σ)_max_ < 0.001
372 parameters	Δρ_max_ = 0.66 e Å^−^^3^
15 restraints	Δρ_min_ = −0.34 e Å^−^^3^

### Special details

**Table:**

Geometry. All e.s.d.'s (except the e.s.d. in the dihedral angle between two l.s. planes) are estimated using the full covariance matrix. The cell e.s.d.'s are taken into account individually in the estimation of e.s.d.'s in distances, angles and torsion angles; correlations between e.s.d.'s in cell parameters are only used when they are defined by crystal symmetry. An approximate (isotropic) treatment of cell e.s.d.'s is used for estimating e.s.d.'s involving l.s. planes.
Refinement. Refinement of *F*^2^ against ALL reflections. The weighted *R*-factor *wR* and goodness of fit *S* are based on *F*^2^, conventional *R*-factors *R* are based on *F*, with *F* set to zero for negative *F*^2^. The threshold expression of *F*^2^ > σ(*F*^2^) is used only for calculating *R*-factors(gt) *etc*. and is not relevant to the choice of reflections for refinement. *R*-factors based on *F*^2^ are statistically about twice as large as those based on *F*, and *R*- factors based on ALL data will be even larger.

### Fractional atomic coordinates and isotropic or equivalent isotropic displacement parameters (Å^2^)

**Table:**

	*x*	*y*	*z*	*U*_iso_*/*U*_eq_	
Fe1	0.448260 (15)	0.23530 (3)	0.15718 (2)	0.02377 (13)	
S1	0.33448 (3)	0.07600 (5)	0.16883 (4)	0.03358 (19)	
N1	0.38927 (10)	0.34694 (15)	0.19124 (14)	0.0311 (5)	
N2	0.49425 (10)	0.36835 (15)	0.12194 (13)	0.0300 (5)	
N3	0.40007 (10)	0.24313 (16)	0.02909 (14)	0.0324 (5)	
N4	0.50712 (9)	0.15827 (15)	0.08713 (13)	0.0280 (5)	
C1	0.33823 (14)	0.3357 (2)	0.2267 (2)	0.0451 (8)	
H1A	0.3242	0.2749	0.2350	0.054*	
C2	0.30476 (15)	0.4114 (2)	0.2520 (2)	0.0542 (9)	
H2A	0.2688	0.4007	0.2762	0.065*	
C3	0.32438 (15)	0.5010 (2)	0.2414 (2)	0.0529 (9)	
H3A	0.3023	0.5518	0.2591	0.063*	
C4	0.37800 (14)	0.5163 (2)	0.20385 (19)	0.0405 (7)	
C5	0.40939 (12)	0.43659 (18)	0.18015 (16)	0.0299 (6)	
C6	0.54657 (13)	0.3774 (2)	0.08885 (18)	0.0374 (7)	
H6A	0.5667	0.3232	0.0742	0.045*	
C7	0.57287 (14)	0.4650 (2)	0.0749 (2)	0.0468 (8)	
H7A	0.6100	0.4688	0.0519	0.056*	
C8	0.54351 (15)	0.5446 (2)	0.0954 (2)	0.0481 (8)	
H8A	0.5603	0.6034	0.0859	0.058*	
C9	0.48812 (14)	0.5384 (2)	0.13080 (18)	0.0390 (7)	
C10	0.46545 (12)	0.44758 (18)	0.14267 (16)	0.0294 (6)	
C11	0.40202 (17)	0.6080 (2)	0.1890 (2)	0.0527 (9)	
H11A	0.3810	0.6612	0.2037	0.063*	
C12	0.45421 (17)	0.6181 (2)	0.1542 (2)	0.0536 (9)	
H12A	0.4687	0.6784	0.1450	0.064*	
C13	0.34567 (14)	0.2827 (2)	0.0008 (2)	0.0483 (8)	
H13A	0.3242	0.3157	0.0372	0.058*	
C14	0.31937 (16)	0.2773 (3)	−0.0807 (2)	0.0602 (10)	
H14A	0.2809	0.3053	−0.0975	0.072*	
C15	0.35054 (17)	0.2305 (3)	−0.1358 (2)	0.0566 (9)	
H15A	0.3335	0.2263	−0.1904	0.068*	
C16	0.40840 (15)	0.1890 (2)	−0.10906 (18)	0.0440 (7)	
C17	0.43098 (13)	0.1969 (2)	−0.02581 (16)	0.0327 (6)	
C18	0.56001 (12)	0.1158 (2)	0.11755 (19)	0.0368 (7)	
H18A	0.5726	0.1188	0.1735	0.044*	
C19	0.59695 (14)	0.0673 (2)	0.0685 (2)	0.0440 (8)	
H19A	0.6337	0.0387	0.0917	0.053*	
C20	0.57940 (15)	0.0616 (2)	−0.0132 (2)	0.0456 (8)	
H20A	0.6040	0.0289	−0.0460	0.055*	
C21	0.52383 (14)	0.1052 (2)	−0.04818 (18)	0.0384 (7)	
C22	0.48900 (12)	0.15288 (18)	0.00512 (16)	0.0308 (6)	
C23	0.44517 (19)	0.1396 (3)	−0.1616 (2)	0.0552 (9)	
H23A	0.4306	0.1343	−0.2169	0.066*	
C24	0.50066 (18)	0.1005 (2)	−0.1325 (2)	0.0524 (9)	
H24A	0.5240	0.0701	−0.1683	0.063*	
O1	0.5000	0.22796 (18)	0.2500	0.0307 (6)	
O1W	0.65938 (13)	0.2776 (2)	0.01141 (19)	0.0760 (8)	
O2	0.39311 (9)	0.13274 (15)	0.17760 (13)	0.0445 (5)	
O2W	0.32992 (16)	0.80292 (19)	0.14393 (18)	0.0765 (8)	
O3	0.31002 (12)	0.0750 (2)	0.08246 (15)	0.0694 (7)	
O3W	0.21915 (15)	0.9578 (3)	0.9884 (2)	0.1018 (11)	
O4	0.35088 (12)	−0.01746 (17)	0.19715 (18)	0.0709 (8)	
O4W	0.26638 (15)	0.1716 (2)	0.36928 (17)	0.0777 (8)	
O5	0.29116 (11)	0.12164 (18)	0.21519 (16)	0.0640 (7)	
H2WA	0.334 (2)	0.792 (3)	0.0943 (11)	0.096*	
H4WA	0.2455 (19)	0.220 (2)	0.352 (2)	0.096*	
H2WB	0.333 (2)	0.8619 (9)	0.151 (2)	0.096*	
H1WA	0.6784 (19)	0.250 (3)	0.0541 (19)	0.096*	
H4WB	0.2867 (18)	0.153 (3)	0.3310 (18)	0.096*	
H1WB	0.6807 (17)	0.3280 (18)	0.008 (3)	0.096*	
H3WB	0.2496 (13)	0.936 (3)	0.966 (3)	0.096*	
H3WA	0.2316 (17)	1.0085 (18)	1.013 (3)	0.096*	

### Atomic displacement parameters (Å^2^)

**Table:**

	*U*^11^	*U*^22^	*U*^33^	*U*^12^	*U*^13^	*U*^23^
Fe1	0.0213 (2)	0.0267 (2)	0.0239 (2)	0.00157 (14)	0.00528 (14)	−0.00084 (15)
S1	0.0288 (4)	0.0339 (4)	0.0391 (4)	−0.0059 (3)	0.0086 (3)	−0.0057 (3)
N1	0.0267 (11)	0.0321 (13)	0.0356 (13)	0.0037 (9)	0.0089 (10)	−0.0025 (10)
N2	0.0285 (12)	0.0313 (12)	0.0305 (12)	0.0006 (9)	0.0054 (9)	0.0018 (10)
N3	0.0249 (12)	0.0375 (13)	0.0338 (13)	−0.0004 (9)	−0.0003 (10)	0.0025 (10)
N4	0.0270 (11)	0.0290 (12)	0.0285 (12)	0.0002 (9)	0.0057 (9)	−0.0043 (9)
C1	0.0353 (16)	0.0445 (18)	0.059 (2)	−0.0010 (13)	0.0179 (15)	−0.0051 (15)
C2	0.0362 (17)	0.061 (2)	0.070 (2)	0.0075 (15)	0.0244 (17)	−0.0127 (19)
C3	0.0414 (18)	0.055 (2)	0.062 (2)	0.0223 (15)	0.0065 (16)	−0.0137 (17)
C4	0.0385 (16)	0.0369 (17)	0.0448 (18)	0.0129 (13)	−0.0001 (14)	−0.0047 (14)
C5	0.0280 (13)	0.0315 (15)	0.0288 (15)	0.0064 (11)	−0.0014 (11)	−0.0006 (11)
C6	0.0326 (15)	0.0409 (17)	0.0402 (17)	−0.0023 (12)	0.0101 (13)	0.0034 (13)
C7	0.0376 (17)	0.055 (2)	0.049 (2)	−0.0118 (15)	0.0106 (14)	0.0064 (16)
C8	0.0517 (19)	0.0401 (18)	0.051 (2)	−0.0160 (15)	0.0028 (16)	0.0063 (15)
C9	0.0460 (17)	0.0319 (15)	0.0370 (16)	−0.0044 (13)	−0.0021 (13)	0.0050 (13)
C10	0.0312 (14)	0.0285 (14)	0.0270 (14)	0.0011 (11)	−0.0021 (11)	0.0011 (11)
C11	0.065 (2)	0.0285 (16)	0.063 (2)	0.0141 (15)	0.0031 (18)	−0.0055 (15)
C12	0.070 (2)	0.0262 (16)	0.063 (2)	−0.0002 (15)	0.0034 (19)	0.0021 (15)
C13	0.0364 (17)	0.060 (2)	0.0479 (19)	0.0023 (15)	0.0028 (14)	0.0070 (16)
C14	0.0385 (18)	0.079 (3)	0.057 (2)	−0.0018 (17)	−0.0150 (17)	0.023 (2)
C15	0.057 (2)	0.071 (2)	0.0373 (18)	−0.0160 (18)	−0.0108 (16)	0.0111 (17)
C16	0.0529 (19)	0.0467 (18)	0.0312 (16)	−0.0169 (15)	0.0009 (14)	0.0044 (14)
C17	0.0367 (15)	0.0324 (14)	0.0291 (15)	−0.0084 (12)	0.0049 (12)	0.0015 (12)
C18	0.0297 (14)	0.0370 (16)	0.0441 (17)	0.0026 (12)	0.0057 (12)	−0.0030 (13)
C19	0.0305 (15)	0.0391 (17)	0.064 (2)	0.0040 (12)	0.0138 (15)	−0.0052 (15)
C20	0.0472 (18)	0.0387 (17)	0.057 (2)	−0.0020 (14)	0.0297 (16)	−0.0101 (15)
C21	0.0473 (17)	0.0331 (15)	0.0386 (17)	−0.0110 (13)	0.0206 (14)	−0.0049 (13)
C22	0.0366 (15)	0.0283 (14)	0.0285 (14)	−0.0067 (11)	0.0083 (12)	−0.0002 (11)
C23	0.081 (3)	0.058 (2)	0.0278 (17)	−0.0188 (19)	0.0112 (17)	−0.0084 (15)
C24	0.074 (2)	0.051 (2)	0.0370 (18)	−0.0136 (18)	0.0265 (17)	−0.0101 (15)
O1	0.0298 (14)	0.0383 (15)	0.0241 (13)	0.000	0.0038 (11)	0.000
O1W	0.0698 (19)	0.077 (2)	0.084 (2)	0.0032 (15)	0.0218 (16)	0.0093 (16)
O2	0.0327 (11)	0.0473 (12)	0.0547 (14)	−0.0119 (9)	0.0102 (10)	−0.0007 (10)
O2W	0.109 (2)	0.0455 (15)	0.076 (2)	−0.0050 (16)	0.0158 (18)	−0.0005 (14)
O3	0.0637 (16)	0.089 (2)	0.0500 (15)	−0.0046 (14)	−0.0119 (12)	−0.0159 (14)
O3W	0.081 (2)	0.119 (3)	0.104 (3)	−0.027 (2)	0.005 (2)	−0.030 (2)
O4	0.0770 (18)	0.0403 (14)	0.094 (2)	−0.0042 (12)	0.0056 (15)	0.0135 (13)
O4W	0.078 (2)	0.096 (2)	0.0598 (18)	0.0219 (16)	0.0127 (15)	−0.0060 (16)
O5	0.0531 (14)	0.0703 (17)	0.0763 (18)	−0.0122 (12)	0.0377 (13)	−0.0198 (14)

### Geometric parameters (Å, °)

**Table:**

Fe1—O1	1.7804 (10)	C9—C12	1.427 (4)
Fe1—O2	1.936 (2)	C11—C12	1.335 (5)
Fe1—N4	2.125 (2)	C11—H11A	0.9300
Fe1—N1	2.151 (2)	C12—H12A	0.9300
Fe1—N3	2.237 (3)	C13—C14	1.393 (5)
Fe1—N2	2.243 (2)	C13—H13A	0.9300
S1—O4	1.435 (3)	C14—C15	1.370 (5)
S1—O5	1.435 (2)	C14—H14A	0.9300
S1—O3	1.456 (3)	C15—C16	1.401 (5)
S1—O2	1.491 (2)	C15—H15A	0.9300
N1—C1	1.321 (4)	C16—C17	1.403 (4)
N1—C5	1.363 (3)	C16—C23	1.431 (5)
N2—C6	1.322 (3)	C17—C22	1.434 (4)
N2—C10	1.349 (3)	C18—C19	1.389 (4)
N3—C13	1.332 (4)	C18—H18A	0.9300
N3—C17	1.360 (4)	C19—C20	1.355 (5)
N4—C18	1.331 (3)	C19—H19A	0.9300
N4—C22	1.362 (3)	C20—C21	1.407 (5)
C1—C2	1.387 (4)	C20—H20A	0.9300
C1—H1A	0.9300	C21—C22	1.401 (4)
C2—C3	1.358 (5)	C21—C24	1.419 (4)
C2—H2A	0.9300	C23—C24	1.351 (5)
C3—C4	1.398 (4)	C23—H23A	0.9300
C3—H3A	0.9300	C24—H24A	0.9300
C4—C5	1.399 (4)	O1—Fe1^i^	1.7804 (10)
C4—C11	1.432 (5)	O1W—H1WA	0.861 (10)
C5—C10	1.436 (4)	O1W—H1WB	0.856 (10)
C6—C7	1.397 (4)	O2W—H2WA	0.849 (10)
C6—H6A	0.9300	O2W—H2WB	0.845 (10)
C7—C8	1.358 (5)	O3W—H3WB	0.848 (7)
C7—H7A	0.9300	O3W—H3WA	0.849 (7)
C8—C9	1.399 (4)	O4W—H4WA	0.851 (10)
C8—H8A	0.9300	O4W—H4WB	0.854 (10)
C9—C10	1.400 (4)		
			
O1—Fe1—O2	97.99 (10)	C9—C8—H8A	119.9
O1—Fe1—N4	94.85 (9)	C8—C9—C10	116.7 (3)
O2—Fe1—N4	97.58 (10)	C8—C9—C12	123.9 (3)
O1—Fe1—N1	98.39 (9)	C10—C9—C12	119.4 (3)
O2—Fe1—N1	96.31 (10)	N2—C10—C9	123.4 (3)
N4—Fe1—N1	159.26 (9)	N2—C10—C5	117.4 (2)
O1—Fe1—N3	168.95 (6)	C9—C10—C5	119.3 (2)
O2—Fe1—N3	88.80 (9)	C12—C11—C4	121.0 (3)
N4—Fe1—N3	75.53 (9)	C12—C11—H11A	119.5
N1—Fe1—N3	89.46 (9)	C4—C11—H11A	119.5
O1—Fe1—N2	91.27 (9)	C11—C12—C9	121.4 (3)
O2—Fe1—N2	168.33 (8)	C11—C12—H12A	119.3
N4—Fe1—N2	88.65 (9)	C9—C12—H12A	119.3
N1—Fe1—N2	75.21 (10)	N3—C13—C14	123.3 (3)
N3—Fe1—N2	83.19 (8)	N3—C13—H13A	118.4
O4—S1—O5	113.16 (17)	C14—C13—H13A	118.4
O4—S1—O3	110.74 (17)	C15—C14—C13	119.5 (3)
O5—S1—O3	110.24 (16)	C15—C14—H14A	120.2
O4—S1—O2	107.19 (15)	C13—C14—H14A	120.2
O5—S1—O2	107.91 (14)	C14—C15—C16	119.2 (3)
O3—S1—O2	107.35 (14)	C14—C15—H15A	120.4
C1—N1—C5	118.0 (2)	C16—C15—H15A	120.4
C1—N1—Fe1	125.6 (2)	C15—C16—C17	117.4 (3)
C5—N1—Fe1	116.24 (17)	C15—C16—C23	123.8 (3)
C6—N2—C10	118.0 (2)	C17—C16—C23	118.8 (3)
C6—N2—Fe1	128.21 (19)	N3—C17—C16	123.5 (3)
C10—N2—Fe1	113.67 (18)	N3—C17—C22	116.9 (2)
C13—N3—C17	117.1 (3)	C16—C17—C22	119.5 (3)
C13—N3—Fe1	129.5 (2)	N4—C18—C19	122.2 (3)
C17—N3—Fe1	113.38 (17)	N4—C18—H18A	118.9
C18—N4—C22	118.3 (2)	C19—C18—H18A	118.9
C18—N4—Fe1	124.80 (19)	C20—C19—C18	120.0 (3)
C22—N4—Fe1	116.94 (17)	C20—C19—H19A	120.0
N1—C1—C2	122.4 (3)	C18—C19—H19A	120.0
N1—C1—H1A	118.8	C19—C20—C21	119.9 (3)
C2—C1—H1A	118.8	C19—C20—H20A	120.1
C3—C2—C1	120.2 (3)	C21—C20—H20A	120.1
C3—C2—H2A	119.9	C22—C21—C20	116.9 (3)
C1—C2—H2A	119.9	C22—C21—C24	119.2 (3)
C2—C3—C4	119.4 (3)	C20—C21—C24	123.9 (3)
C2—C3—H3A	120.3	N4—C22—C21	122.8 (3)
C4—C3—H3A	120.3	N4—C22—C17	117.2 (2)
C3—C4—C5	117.2 (3)	C21—C22—C17	120.0 (3)
C3—C4—C11	123.7 (3)	C24—C23—C16	121.4 (3)
C5—C4—C11	119.1 (3)	C24—C23—H23A	119.3
N1—C5—C4	122.8 (3)	C16—C23—H23A	119.3
N1—C5—C10	117.3 (2)	C23—C24—C21	121.0 (3)
C4—C5—C10	119.8 (3)	C23—C24—H24A	119.5
N2—C6—C7	122.8 (3)	C21—C24—H24A	119.5
N2—C6—H6A	118.6	Fe1^i^—O1—Fe1	173.30 (17)
C7—C6—H6A	118.6	H1WA—O1W—H1WB	103 (2)
C8—C7—C6	119.1 (3)	S1—O2—Fe1	157.16 (15)
C8—C7—H7A	120.5	H2WA—O2W—H2WB	107 (2)
C6—C7—H7A	120.5	H3WB—O3W—H3WA	107.4
C7—C8—C9	120.1 (3)	H4WA—O4W—H4WB	107 (2)
C7—C8—H8A	119.9		

Symmetry codes: (i) −*x*+1, *y*, −*z*+1/2.

### Hydrogen-bond geometry (Å, °)

**Table:**

*D*—H···*A*	*D*—H	H···*A*	*D*···*A*	*D*—H···*A*
O3W—H3WA···O3^ii^	0.85 (1)	2.14 (3)	2.872 (4)	144 (4)
O2W—H2WB···O4^iii^	0.85 (1)	1.89 (2)	2.713 (4)	163 (4)
O4W—H4WB···O5	0.85 (1)	1.98 (2)	2.756 (4)	151 (4)
O1W—H1WB···O3W^iv^	0.86 (1)	2.06 (1)	2.909 (5)	171 (4)
O1W—H1WA···O4W^i^	0.86 (1)	1.97 (2)	2.811 (5)	165 (5)
O3W—H3WB···O4W^v^	0.85 (1)	2.28 (3)	2.964 (5)	138 (3)

Symmetry codes: (ii) *x*, *y*+1, *z*+1; (iii) *x*, *y*+1, *z*; (iv) *x*+1/2, *y*−1/2, *z*−1; (i) −*x*+1, *y*, −*z*+1/2; (v) *x*, −*y*+1, *z*+1/2.
